# An *In Vitro* Method for Studying the Three-Way Interaction between Soybean, *Rhizophagus irregularis* and the Soil-Borne Pathogen *Fusarium virguliforme*

**DOI:** 10.3389/fpls.2017.01033

**Published:** 2017-06-16

**Authors:** María L. Giachero, Nathalie Marquez, Adrien Gallou, Celina M. Luna, Stéphane Declerck, Daniel A. Ducasse

**Affiliations:** ^1^Instituto de Patología Vegetal – Centro de Investigaciones Agropecuarias, Instituto Nacional de Tecnología AgropecuariaCórdoba, Argentina; ^2^Consejo Nacional de Investigaciones Científicas y TécnicasCórdoba, Argentina; ^3^Centro Nacional de Referencia de Control BiológicoTecomán, Mexico; ^4^Instituto de Fisiología y Recursos Genéticos Vegetales, Centro de Investigaciones Agropecuarias – Instituto Nacional de Tecnología AgropecuariaCórdoba, Argentina; ^5^Earth and Life Institute, Applied Microbiology, Mycology, Université catholique de LouvainLouvain-la-Neuve, Belgium

**Keywords:** arbuscular mycorrhizal fungi, *Fusarium virguliforme*, *in vitro* system, soybean, plant–microbe interaction

## Abstract

In this work, we described an *in vitro* system adequate for investigating the pathosystem soybean/arbuscular mycorrhizal fungi (AMF)/*Fusarium virguliforme.* Pre-mycorrhized plantlets with *Rhizophagus irregularis* were infected by *F. virguliforme* either locally via a plug of gel supporting mycelium (Method 1) or via a macroconidia suspension applied to the medium surface (Method 2). Root colonization by the AMF and infection by the pathogen were similar to the usual observations in pot experiments. Within a period of 18 days, more than 20% of the roots were colonized by the AMF and infection by the pathogen was observed in all the plants. In presence of AMF, a decrease in symptoms and in the level of root tissue infection was noticed. With Method 1, smaller necrotic lesions were observed in the pre-mycorrhized plantlets. In Method 2, pathogen infection was slower but more homogenous. These results demonstrated the suitability of the *in vitro* cultivation system to study the pathosystem soybean/AMF/*F. virguliforme*. We propose this *in vitro* cultivation system for studying the mechanisms involved in the biocontrol conferred by AMF against *F. virguliforme* in soybean.

## Introduction

Studies on the belowground plant–microbes interaction have increased tremendously in number and complexity in recent years (Brink, 2016). During the last decade, this increasing interest resulted in the development of several innovative techniques to investigate the intricate rhizosphere community (Oburger and Schmidt, 2016). Soil is now viewed as a complex universe where plants interact with a wide range of microbes and minerals, resulting in a true system where nothing can be modified without affecting everything else (Young and Crawford, 2004). Many of the microorganisms that are normally present in the soil, actually inhabit the rhizosphere and interact with plants. Those plant–microorganisms interactions may be beneficial or harmful. Among the first are the arbuscular mycorrhizal fungi (AMF). These soil fungi have been reported to improve plant resistance/tolerance to pests and diseases (Gutjahr and Parniske, 2013). AMF are obligate root symbionts that rely on their host for their carbohydrates and as a consequence, it is impossible to grow them in axenic conditions, i.e., independently from a suitable host plant. Nevertheless, in the last decades, a number of cultivation systems have been developed to grow them *in vitro* with root organs (Gutjahr and Parniske, 2013) or whole plants (Voets et al., 2005, 2009; Dupré de Boulois et al., 2006; Koffi et al., 2009). These *in vitro* systems have allowed the in-deep study of the plant-AMF symbiotic association (Koffi et al., 2013).

Soil borne pathogens represent a threat to agriculture generating important yield losses, depending upon the pathogen and the crop (Oerke, 2006). One example is the “Sudden Death Syndrome” (SDS), a severe disease in soybean caused by a complex of at least four species of *Fusarium* sp., among which *Fusarium virguliforme* and *F. tuccumaniae* are the most prevalent in Argentina (O’Donnell et al., 2010).

*Fusarium virguliforme* causes root rot as well as vascular discoloration of roots and stems. Root infection is often accompanied by foliar symptoms characterized, in the early stages of infection, by interveinal chlorosis followed at the later stages by necrosis and, in the most severe cases, by flower and pod abscission (Rupe, 1989) and defoliation (Genre et al., 2005). In the worst situations, SDS can cause up to 90% yield losses has observed in some areas of Argentina (Genre et al., 2005). Symptoms may be more severe on leaves, but reduction in yield is mostly attributed to both root infection and foliar symptoms (Rupe et al., 1989; Genre et al., 2008).

Recently, Bressano et al. (2010) developed an *in vitro* system to infect soybean plantlets with *Macrophomina phaseolina*, a fungal soil-borne pathogen. This system allowed the study of the pre-penetration and penetration process of the pathogen within the roots as well as the early responses of the host plant to infection. Similarly, the tripartite interactions, involving a mycorrhizal plant and a leaf pathogen (potato/AMF with the oomycete *Phytophthora infestans*) (Gallou et al., 2011) or nematodes (banana/AMF with the nematode *Radopholus similis)* (Koffi et al., 2013) and even with beneficial microorganisms [e.g., barrel medic/AMF with *Trichoderma harzianum* (De Jaeger et al., 2011)] have been developed. Gallou (2011) also succeeded in the tripartite association between mycorrhizal potato plantlets and the root pathogen *Rhizoctonia solani*. Using this model, the author was able to investigate the molecular mechanisms involved in the increased resistance of the pre-mycorrhized potato plantlets to this soilborne pathogen.

*In vitro* cultivation systems offers a number of advantages over pot culture experiments which are the absence of unwanted contaminants and the possibility for highly controlled and non-destructive dynamical observations of the interaction between AMF and pathogens (Declerck et al., 2005).

At present, there is no described methodology for the *in vitro* three-way interaction between soybean plantlets, an AMF and a soil-borne fungus. The development of such a system would facilitate comprehension of the mechanisms involved in the increased resistance to *F. virguliforme* in AMF-colonized soybean plantlets.

In the present study we reported and described, for the first time, an *in vitro* cultivation system associating pre-mycorrhized soybean plantlets infected by *F. virguliforme*. We also monitored and compared the early steps of *F. virguliforme* infection progress in presence/absence of the AMF and proposed some reasons to the differences observed.

## Materials and Methods

### Biological Material

A strain of *Rhizophagus irregularis* (Błaszk., Wubet, Renker & Buscot) C. Walker & A. Schüßler as [‘irregulare’] MUCL 41833 was provided by the Glomeromycota *in vitro* collection^1^. The AMF was grown in association with Ri T-DNA transformed carrot (*Daucus carota* L.) roots clone DC1 on Petri plates (90 mm diameter) containing the modified Strullu-Romand (MSR) medium (Declerck et al., 1998) solidified with 3 g L^-1^ Phytagel (Sigma–Aldrich, St. Louis, United States), following the method detailed in (Cranenbrouck et al., 2005). The Petri plates were incubated in the dark in an inverted position at 27°C during several months until thousands of spores were obtained.

A strain of *F. virguliforme* O’Donnell & T. Aoki MUCL 53605, originally isolated from soybean (*Glycine max*) in Argentina (Buenos Aires, San Pedro) (Aoki et al., 2005) was supplied by the Mycothèque de l’Université catholique de Louvain (MUCL^2^). A plug of gel containing several macroconidia and mycelium was placed on 50 ml Potato Dextrose Agar (PDA) (Scharlau Chemie S.A., Barcelona, Spain) in Petri plates (90 mm diameter). The Petri plates were incubated at 25°C in the dark for 7 days.

Seeds of *Medicago truncatula* Gaertn. cv. Jemalong A 17 (SARDI, Australia) were surface-disinfected by immersion in calcium hypochlorite (3.5% active calcium) for 12 min, rinsed three time in deionized sterilized (121°C for 15 min) water and germinated in groups of 15 on Petri plates (90 mm diameter) filled with 35 ml MSR medium without sucrose and vitamins, and solidified with 3 g L^-1^ Phytagel. The Petri plates were incubated at 27°C in the dark for 4 days and subsequently exposed to light for 1 day, before use.

Seeds of soybean [*G. max* (L.) Merr.] cv DON MARIO 4800 were surface-disinfected by immersion in ethanol 70% for 1 min, followed by calcium hypochlorite (10% active calcium) for 2 min, rinsed three time in deionized sterilized (121°C for 15 min) water and germinated in groups of 6 on Petri plates (145 mm diameter) filled with 60 ml MSR medium without sucrose and vitamins, and solidified with 3 g L^-1^ Phytagel. The Petri plates were incubated at 27°C in the dark for 4 days, after exposition to light [average photosynthetic photon flux (PPF) of 225 μmol m^-2^ s^-1^] for 24 h.

### Experimental Set Up

**FIGURE 1:**
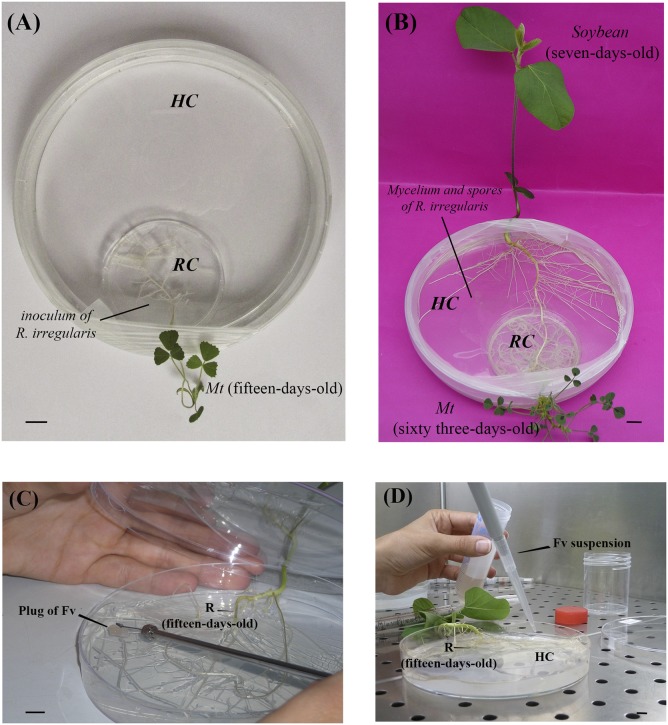
Mycorrhizal Donor Plant (MDP) *in vitro* culture system for the mycorrhization of *Medicago truncatula* (*Mt*) and subsequent infection by *Fusarium virguliforme* [adapted from Voets et al. (2009) and Anene et al., 2013]. **(A)** Bi-compartmented *in vitro* culture system with a root compartment (RC) and a hyphal compartment (HC). A plant of *M. truncatula* (15-days-old) is inserted in the RC and associated to the arbuscular mycorrhizal fungus (AMF) *Rhizophagus irregularis* MUCL 41833. Bar = 1 cm. **(B)** Soybean plantlet (7-days-old) inserted in the HC, with the roots in direct contact with the extraradical mycelium of *R. irregularis* – Bar = 1 cm. **(C)** Root (R) of soybean plants (15-days-old) infected with a plug of Potato Dextrose Agar (PDA) (25 mm^2^) covered with *F. virguliforme* (method 1). Bar = 1 cm. **(D)** Roots of *s*oybean plantlets (15-days-old) infected with a 2 ml suspension of 1.52 × 10^6^ macroconidia/ml of *F. virguliforme* (method 2) – Bar = 1 cm.

The Mycelium Donor Plant (MDP) *in vitro* culture system developed by Voets et al. (2009) and adapted by Anene et al. (2013) for banana was used. Briefly, the cover of a 55 mm diameter Petri plate (named root compartment, RC) was introduced in the base of a 145 mm diameter Petri plate (named hyphal compartment, HC) (**Figure 1A**). The RC and HC were filled with 20 and 100 ml MSR medium lacking vitamins and sucrose and solidified with 3 g L^-1^ Phytagel, respectively. The RC was leaned to the border of the HC and a small opening (±2 mm diameter) was made in the base and the lid of the 145 mm diameter Petri plate. One 4-day-old *M. truncatula* seedling was transferred in the RC with the roots plated on the MSR medium and shoot extending outside the Petri plate via the hole. A plug (9 mm × 5 mm) of MSR medium containing ± 100 spores of *R. irregularis* MUCL 41833 was placed in the vicinity of the roots. The system was then sealed with Parafilm (Pechiney, Plastic Packaging, Chicago, IL, United States) and the hole plastered with sterilized (121°C for 15 min) silicon grease (VWR International, Belgium) to avoid contaminations (**Figure 1A**). The systems were transferred in a growth chamber set at 20/18°C (day/night), 70% relative humidity, with a photoperiod of 16 h d^-1^ and a PPF of 300 μmol m^-2^ s^-1^. Except from the aerial part of *M. truncatula* plants, the rest of the system was covered with an opaque plastic bag to keep the AMF and *M. truncatula* roots in the dark.

After 6 weeks of culture, the mycelium crossed the plastic barrier separating the RC from the HC and a profuse extra-radical mycelium (ERM) network bearing numerous spores developed in the HC. Two weeks later, a new hole was made in the base and the lid of the systems, at the opposite side of *M. truncatula*, to allow the insertion of a 5-days old soybean plantlet with the roots in direct contact with the ERM and shoot extending outside the Petri plate (**Figure 1B**). Simultaneously soybean plantlets were inserted in the MDP *in vitro* culture systems as above but in the absence of AMF (i.e., the control). The systems were sealed carefully and incubated horizontally in a growth chamber under the same conditions as above (with an opaque plastic bag to keep the roots in the dark).

### Inoculation Method and Sampling

After 2 weeks of contact between the soybean roots and the ERM, the RC (i.e., the cover of the 55 mm diameter Petri plate containing the *M. truncatula* plantlet) was removed and replaced by 25 ml MSR medium without sucrose and vitamins and solidified with 3 g L^-1^ Phytagel. At that moment, the systems were inoculated with either a plug of PDA (25 mm^2^) covered by the pathogen (referred as Method 1 – **Figure 1C**) or with 2 ml suspension of 1.52 × 10^6^ macroconidia ml^-1^ in sterile water (referred as Method 2 – **Figure 1D**). In both inoculation methods, the systems were divided randomly into four treatments, each with three replicates: pre-mycorrhized soybean plantlets with/without *F. virguliforme* (+AMF+Fv, +AMF-Fv) and non-mycorrhizal soybean plantlets with/without *F. virguliforme* (-AMF+Fv, -AMF-Fv).

Roots were harvested 48 h after contact of the first hyphae of the pathogen with the root surface (i.e., 96 h after inoculation) for Method 1 or after 72 h post-inoculation for Method 2. Colonization by *R. irregularis* and damage caused by the pathogen were subsequently assessed.

### Root and Leaves Symptoms Evaluation in *F. virguliforme* Infected Plantlets

Symptoms on roots and leaves were monitored just before plantlets were harvested (i.e., 96 h and 72 h after inoculation by the pathogen for Method 1 and 2, respectively).

The evolution of root necrosis differed according to the pathogen inoculation method. In Method 1, the size of the necrotic area at the point where the hyphae of the pathogen contacted the root surface was monitored and compared between mycorrhizal and not mycorrhizal plantlets. In Method 2, the entire root system was observed at 24, 48, and 72 h. Independently of the method of inoculation with the pathogen, the symptoms on leaves were also visually monitored.

### Assessment of Root Colonization by *R. irregularis* and Infection by *F. virguliforme*

Root colonization by AMF was estimated 96 h (Method 1) and 72 h (Method 2) after inoculation by the pathogen. Within such short period it was estimated that the pathogen did not impact the root colonization parameters of the AMF and consequently no colonization analysis was conducted prior to infection by the pathogen.

Roots were cleared in 10% KOH at room temperature for 3 h, rinsed with distilled water, bleached and acidified with HCl 1% and stained with Trypan blue 0.2% at room temperature for 15 min. The percentage of root colonization was subsequently estimated according to McGonigle et al. (1990). Two hundred intersections were observed under a compound microscope (Olympus SZ40, Olympus Optical GmbH, Hamburg, Germany) at 10–40× magnification. Total root colonization (%RC), abundance of arbuscules (%A), and intraradical spores/vesicles (%V) were determined. At each intersection, a mark was made if the vertical crosshair crosses an arbuscule, vesicle or hyphae. Intersections were counted in the following categories: “negative” (i.e., no AM fungus observed in root), “hyphae only,” “arbuscules” and “vesicles.” If the vertical crosshair crossed one or more arbuscules or vesicles, the category was incremented by one, and similarly for intersections where hyphae only were observed. When both arbuscules and vesicles were visualized at an intersection, the total number of intersections was only added by one. The arbuscular colonization and vesicles colonization were calculated by dividing the count for the “arbuscules” and “vesicles” categories, respectively, by the total number of intersections examined. Hyphal colonization was calculated as the proportion of non-negative intersections.

Infection by *F. virguliforme* was confirmed under the microscope (Nikon labophot-2, Japan) at 10–40× magnification and the estimation of root infection was done following staining with Trypan blue 0.2% as above. The estimation of the level of roots infection by *F. virguliforme* was done following the methodology described by McGonigle et al. (1990) slightly modified. For each treatment, 10 mm long root pieces were selected randomly, mounted in glycerine on microscope slides and covered with 40 × 22 mm coverslips. Between 2 and 4 slides, each covered with 10 pieces of roots, were used per replicate. Roots were aligned parallel to the long axis of the slide and observed as follows under a magnification of 40×: to examine each intersection, the plane of focus was moved completely through the root and whether the vertical crosshair actually cuts any hyphae, were considered like positive. Intersections were counted in the following categories; ‘absence of hyphae’ (no fungal material in root), and ‘presence of hyphae.’ Hyphal colonization was calculated as the proportion of non-negative intersections.

When pre-mycorrhized *F. virguliforme* infected roots were examined, both mycelia were clearly distinguished by their morphology and growing pattern. While *R. irregularis* grew displaying an intercellular net-like pattern with well-stained cell walls, the pathogen showed a thinner and more transparent hyphae and straighter and linear growing pattern. The percentage of infected roots was estimated as the ratio between infected roots and total number of roots examined.

To determine whether the cells of a brownish tissue was dead or not, an FDA-PI staining was performed on detached roots (see Supplementary Material).

### Statistical Analysis

The experiment was repeated twice. Percentages of root colonization by AMF and root infection by *F. virguliforme* were tested for normal distribution. Data were subsequently subjected to the LSD Fisher’s honest significant difference (HSD) test in order to identify the significant differences (*P* ≤ 0.05) between treatments. Data analysis was performed with the statistical package INFOSTAT (Di Rienzo et al., 2011).

## Results

### Root Colonization by the AMF

Root colonization by the AMF was estimated 96 h (Method 1) and 72 h (Method 2) after inoculation by *F. virguliforme.* This corresponded to 19 (Method 1) and 18 (Method 2) days post-plating of the soybean plantlets in the HC containing the ERM of the AMF. The non-mycorrhizal controls were similarly harvested and observed.

Intraradical hyphae, arbuscules and vesicles/spores were observed in the +AMF-Fv and +AMF+Fv treatments. In general, no significant differences were observed in %A or %V between the +AMF-Fv and +AMF+Fv treatments (**Table 1**) unless in one of the %RC. No root colonization was noticed in the non-mycorrhizal controls (-AMF+Fv and -AMF-Fv).

**Table 1:** Root colonization of soybean plants plated on actively growing extraradical mycelium networks of *Rhizophagus irregularis* in presence (+AMF+Fv) or absence (+AMF-Fv) of the pathogen *Fusarium virguliforme*.

Treatment	%RC	%A	%V
+AMF-Fv	22.93 ± 5.57^a^	6.68 ± 2.36^a^	3.33 ± 1.44^a^
+AMF+Fv	24.97 ± 5.76^a^	4.50 ± 2.36 ^a^	0,17 ± 1.44 ^a^
+AMF-Fv	40.97 ± 2.71 ^a^	21,85 ± 2.81 ^a^	2.62 ± 1.11^a^
+AMF+Fv	51.33 ± 3.03 ˆb	16.64 ± 3.14 ^a^a	2.33 ± 1.25 ^a^

*For each structure (i.e., %RC, %A, and %V), values (mean ± standard error of four replicates) followed by identical letter do not differ significantly (p ≤ 0.05).*

### Infection with *F. virguliforme*

**FIGURE 2:**
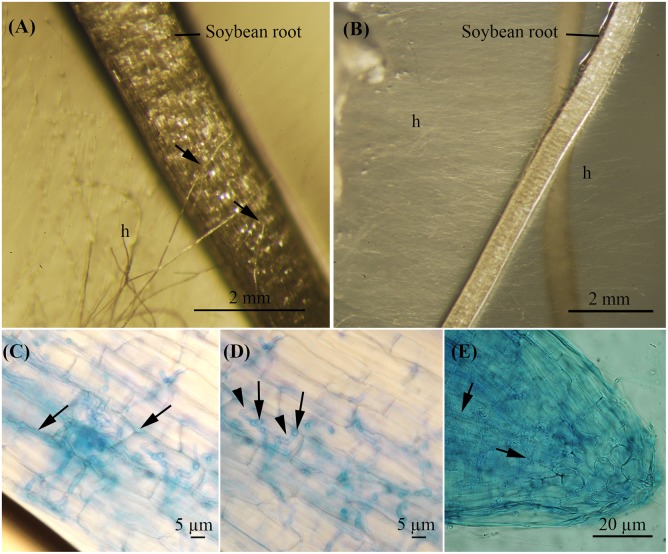
Roots from soybean plantlets cultivated *in vitro* in the MDP *in vitro* culture system showing early infection stages by *F. virguliforme* observed under the stereo microscope **(A,B)** and under the microscope **(C–E)**. **(A)** Fungal hyphae contacting the host root surface 2 days after inoculation of the pathogen (soybean plantlet of 17-days-old). Bar = 2 mm. **(B)** Hyphal development 4 days after inoculation of the pathogen (soybean plantlet of 19-days-old). Bar = 2 mm. **(C–E)** Soybean roots stained with Trypan blue 0.2%. **(C)** Intercellular growth of *F. virguliforme* hyphae. Bar = 5 μm. **(D)** Swollen and pigmented invasive structures (black arrow), intracellular growth of hyphae (arrowhead). Bar = 5 μm. **(E)** Hyphae in the tip roots zone. Bar = 20 μm.

In Method 1 (inoculation with a plug of PDA supporting mycelium), hyphae of *F. virguliforme* reached the surface of the roots 2 days after inoculation, independently of the presence/absence of AMF associated to the soybean plantlets. Following contact, the pathogen developed profusely from a few hyphae (**Figure 2A**) to a dense network of hyphae during the following 48 h (**Figure 2B**). Pathogen roots penetration and subsequent root tissues invasion was confirmed by microscopic observation. Careful examination of *F. virguliforme* within the roots revealed hyphae growing inter and intracellularly. Intercellular growth was predominantly parallel to the root axis (**Figure 2C**), whereas intracellular hyphae was perpendicular to it (**Figure 2D**). Many hyphae were also observed in the root tip zone (**Figure 2E**). Fungal structures with a swollen and pigmented cell wall were evidenced (**Figures 2C,D**).

In Method 2 (inoculation with macroconidia) a faster and denser development of hyphae was noticed on the surface of the MSR medium. Similarly to the inoculation via Method 1, hyphae and infective fungal structures were observed in the roots extending inter and intracellularly. The whole root system was infected by the pathogen irrespective of the treatment. Nevertheless, the percentage of root infection was significantly lower (*p* = 0.0129) in the +AMF+Fv treatment as compared to the -AMF+Fv treatment (**Table 2**).

**Table 2:** Percentage of root infection in presence (+AMF+Fv) or absence (-AMF+Fv) of the mycorrhizal fungi.

Treatment	Root infection (%)
+AMF+Fv	35.7% ± 1.7 a
-AMF+Fv	44.0% ± 2.9 b

*Values (mean ± standard error of 4 replicates) followed by identical letter do not differ significantly (p ≤ 0.05).*

**FIGURE 3:**
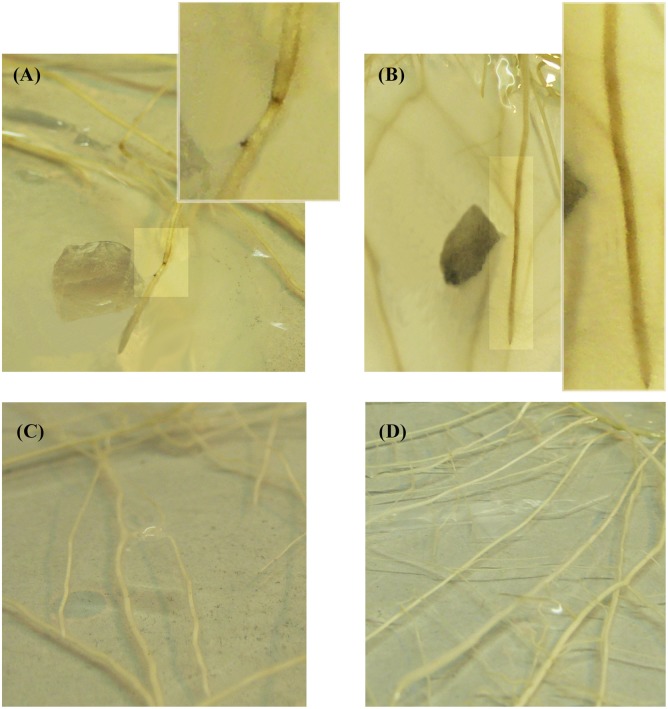
Roots from soybean plantlets cultivated *in vitro* in modified Strullu-Romand (MSR) medium observed by naked eyes. **(A)** pre-mycorrhized soybean plantlets inoculated with a plug of PDA (25 mm^2^) covered by *F. virguliforme* (+AMF+Fv). The white box shows an enlarged picture of root necrotic symptoms from **A**, 96 h following inoculation. **(B)** Non-mycorrhizal soybean plantlets inoculated with a plug of PDA (25 mm^2^) covered by *F. virguliforme*. The white box shows an enlarged picture of root necrotic symptoms from **B**, 96 h following inoculation. **(C)** pre-mycorrhized soybean plantlets without *F. virguliforme* (+AMF-Fv). **(D)** Non-mycorrhizal soybean plantlets without *F. virguliforme* (–AMF-Fv).

Necrotic areas were observed on the surface of the roots of the plantlets in both treatments, -AMF+Fv and +AMF+Fv, and with both inoculation method. In Method 1, small necrotic areas of about 1.3 mm diameter were noticed near the penetration point of the pathogen, in the soybean plantlets of the +AMF+Fv treatment (**Figure 3A**). To the contrary, in the plantlets of the -AMF+Fv treatment, *F. virguliforme* developed a necrotic area on either side of the infection point with a diameter of about 20 mm (˜95% higher) (**Figure 3B**). No symptoms were detected in roots of pre-mycorrhized soybean plantlets without *F. virguliforme* (+AMF-Fv) (**Figure 3C**) and non-mycorrhizal soybean plantlets without *F. virguliforme* (-AMF-Fv) (**Figure 3D**). State of cells viability was assessed by FDA-PI staining. (Supplementary Material).

The evaluation of leaf symptoms was performed 96 and 72 h after inoculation by *F. virguliforme*. No symptoms were observed on the leaves of any soybean plantlets independently of the presence/absence of AMF and of the pathogen inoculation method.

## Discussion

In this study we reported for the first time on the tripartite interaction between soybean, a root symbiont (the AMF *R. irregularis* MUCL 41833) and a major root pathogen (*F. virguliforme* MUCL 53605) under *in vitro* culture conditions. Both microorganisms colonized the plantlet roots and the triple interaction was monitored by direct observation and under the microscope. The pathogen was inoculated following two methodologies: using a plug of gel covered by the pathogen (Method 1) and using a suspension of macroconidia (Method 2). In both cases, the pathogen was able to establish infection in the root tissues. This was evaluated under the microscope by determining the percentages of root infection by the pathogen. The same approach was followed to evaluate the level of root colonization by the AMF.

Root organ cultures colonized by AMF have been used to study mycorrhizal interaction for many years (Fortin et al., 2002; Elsen et al., 2003), while the interaction with whole plants is more recent (Voets et al., 2005; Dupré de Boulois et al., 2006; Koffi et al., 2009; Voets et al., 2009). For instance, Gallou et al. (2011) investigated the tripartite interaction between pre-mycorrhized potato plantlets and a leaf pathogen, *P. infestans*. Finally, Anene et al. (2013) investigated the interaction between pre-colonized banana plantlets and the nematode *R. similis*. Bressano et al. (2010) monitored the development of *M. phaseolina* in soybean roots under *in vitro* conditions. These authors demonstrated not only that the pathogen under study was able of infecting the host roots but also that the infecting structures observed *in vitro* and *in vivo* were similar. Therefore these systems paved the way for the tripartite interaction study between soybean, AMF and *F. virguliforme*.

In the present study, two inoculation methods were tested and compared. Both methods were effective, and 100% of the plantlets were infected within 3 days. However, the infection process differed slightly between the two methods. With Method 1, the number of infection sites was limited and almost restricted to the area where the plug supporting the pathogen was placed. Hyphae extending form the plug contacted the neighboring roots and developed localized symptoms. Conversely, with Method 2, infection was almost homogenous with multiple infection units distributed on the whole root system. Method 1 seems thus, particularly adapted for short-term time-course infection studies since the development of the pathogen can be easily and accurately monitored. This inoculation method is suitable for performing studies on dynamic of defense genes expression. Following the same rationale, studies on induced systemic resistance (ISR) or mycorrhizal induced resistance (MIR), generally conducted in tri-partite systems separating roots infected by a pathogen from non-infected roots (Khaosaad et al., 2007), can also be performed with this method. Besides, our methodology is feasible to be applied with a high-throughput system analysis like RNA sequencing (RNA-seq), DNA microarrays or even a chemical library.

Method 2, on the other hand, is more adapted when a high level of infection in whole root system is requested representing an infection shock for the plant.

Root symptoms were observed in the mycorrhizal as well as non-mycorrhizal plantlets. However, in the pre-mycorrhized plantlets infected by Method 1, the size of the necrotic lesion on the roots was approximately 95% smaller as compared to the non-mycorrhizal plantlets. Similarly, the level of necrosis on the whole root system was less intense in the pre-mycorrhized plantlets infected by Method 2. No symptoms were evidenced on the leaves. This was probably related to the short-term infection study (96 and 72 h for inoculation Method 1 and 2, respectively).

Interestingly, when the presence of the pathogen inside the root tissue was quantified, the amount of intraradical hyphae of the pathogen was significantly lower in the pre-mycorrhized plantlets as compared to the non-mycorrhizal ones. These observations support the hypothesis that early AMF root colonization, i.e., prior to any interaction with the pathogen, increases the ability of the colonized plantlet to withstand the competition with the pathogen. This hypothesis was supported by numerous studies reporting reduction of severity in diseases caused by soil-borne and above-ground pathogens in pre-mycorrhized plants (Whipps, 2004; Pozo et al., 2009).

These results, demonstrated the suitability of the *in vitro* cultivation system to study the tripartite interaction between soybean, an AMF (*R. irregularis*) and a major root pathogen (*F. virguliforme*). It offers large perspectives to investigate the mechanisms involved in early and transient protection conferred to plants in presence of the symbiotic fungus. In particular, molecular studies [e.g., defense gene expression analysis (Gallou et al., 2011)] and microarray approaches (Gallou et al., 2012) would benefit from such system.

The main innovations of the system here presented compared to other similar systems developed previously by other authors are referred to the type of pathogen, a double colonization of the same plant organ by two microorganism and the verification of this fact and to corroborate symptoms attenuation in an *in vitro* system. In other words, we worked with a soil-borne pathogen that infects roots. This implies not only the use of a different pathogen not previously reported, but also that the pathogen develops in a different environment such as the soil, which is not a minor fact and has not been reported previously. On the other hand, infection by the pathogen and colonization by the mycorrhizal fungus in the roots was verified, this is to say, a complex interaction system was established in the same organ of the plant, the roots. And finally, it is the first time that symptoms attenuation is reported in an *in vitro* mycorrhizal system and although it is true that this symptoms mitigation was estimated qualitatively, it opens the possibilities to use this system to perform more exhaustive studies.

However, the system also have some limitations which most important is the limited space for the protagonists to develop. Indeed the *in vitro* cultivation system is not adapted for long-term interaction studies due to the fast growth of the soybean plantlet as well as the pathogen on the synthetic growth medium. The use of the autotrophic *in vitro* cultivation system represents a substantial improvement with regard to root organ cultures often used in interaction studies of AMF with pathogens, mainly because of the presence of photosynthetic active tissues and the absence of sugar and vitamins in the growth medium. This model system offers a highly controlled option to investigate the molecular (e.g., defense gene expression) as well as other mechanisms involved in the bio-protection conferred by AMF to a major soil-borne pathogen of soybean.

## Author Contributions

MG and NM evenly contributed to the manuscript in experiment developing and writing. AG contributed with experiment developing and critical review, CL contributed with critical views, SD and DD contributed in experimental planning and writing.

## Conflict of Interest Statement

The authors declare that the research was conducted in the absence of any commercial or financial relationships that could be construed as a potential conflict of interest.

## Footnotes

1 http://www.mycorrhiza.be/ginco-bel

2 http://bccm.belspo.be/about/mucl.php
