# Supplementary material for: An In Vitro Method for Studying the Three-Way Interaction between Soybean, Rhizophagus irregularis and the Soil-Borne Pathogen Fusarium virguliforme
Source: Front Plant Sci. 2017 Jun 16;8:1033. doi: 10.3389/fpls.2017.01033 (PMC5472683; doi:10.3389/fpls.2017.01033)
Supplement: Supplementary file 1 [file Data_Sheet_1.docx]

**Supplementary Material**

**Cell viability assay.**

**Materials and methods**

Seeds of soybean [Glycine max (L.) Merr.] cv DON MARIO 4800 were surface disinfected by immersion in ethanol 70% for 1 min, followed by calcium hypochlorite (10% active calcium) for 2 min, rinsed three time in deionized sterilized (121°C for 15min) water and germinated in groups of 6 on Petri plates (145 mm in diam.) filled with 60 ml MSR medium without sucrose and vitamins, and solidified with 3 g L-1 Phytagel. The Petri plates were incubated at 27°C in the dark for 4 days, after exposition to light (average photosynthetic photon flux (PPF) of 225 μmol m−2 s−1) for 24 h.

Sterile plastic Petri dishes (145 mm ø) were filled with 100 ml MSR medium lacking vitamins and sucrose and solidified with 3 g L-1 Phytagel^©^. A hole was made in the base and the lid of the Petri dish to allow the insertion of a 5-days old soybean plantlet with the roots in direct contact with the media, and the leaves extending outside the Petri plate. The systems were sealed carefully and incubated horizontally (covered by an opaque plastic bag to keep the roots in the dark) in a growth chamber set at 20/18°C (day/night), 70 % relative humidity, with a photoperiod of 16 h d^-1^ and a PPF of 300 μmolm^−2^s^−1^.

After two weeks, the systems were divided randomly into two treatments, each with three replicates: with (+Fv) and without pathogen (-Fv). The systems were inoculated with either a plug of PDA (25 mm²) or with a plug of PDA (25 mm²) covered by the pathogen (referred as Method 1 – Fig. 1C in the manuscript).

Root areas near to the infection point of the pathogen were observed at naked eye, and root fragments with and without symptoms were sampled (Figure 1A).

Viability of root fragments was determined by the fluorescein diacetate–propidium iodide staining (FDA–PI) method described by Z. Xia, 1995. Fluorescein diacetate (FDA) and propidium iodide (PI) stain viable cells and dead cells, respectively. Non-fluorescent FDA molecules pass through the intact plasma membrane and are hydrolyzed by intracellular esterases into fluorescein, exhibiting green fluorescence in the cytoplasm. In contrast, PI cannot pass through a viable cell membrane. PI reaches the nucleus by passing through a disrupted plasma membrane of dead cell and intercalates with DNA to form a bright red fluorescent complex in the nucleus. This is to say that that PI preferentially labels nuclei of dead cells. In brief, the roots were incubated for 25 min at room temperature in final volume of 2ml of a solution containing: 2μl of PI solution (stock solution 2 mg/ml) plus 2μl of FDA solution (stock solution 0.5% w/v) in ultrapure water. Then the root fragments were observed under a Nikon Eclipse Ti, laser scanning confocal microscope. Fluorescein was excited using the 488 nm line of an Argon laser and emission collected through a 515/30nm BA filter. PI was excited using a 543 nm He-Ne laser and emission collected with a 605/75nm BA filter. Images of 1024 dpi were acquired with the EZC1(ver.3.01) software using 6ms of dwelling time and the same gain in every detector.

**Results.**

To ensure that there was no overlap in fluorescence detection between FDA and PI, it was performed first individual staining with each dye and imaged with detection configured for both. FDA staining alone produced no detectable red fluorescence, while PI staining alone produced no detectable green fluorescence (data not shown). These results indicate that green fluorescence was specific to fluorescein, red fluorescence was specific to PI, and that auto-fluorescence was not being collected in either channel.

To evaluate the cell viability, fragment roots were sampled 72 h post-inoculation, when symptoms of apparent necrosis, this is brownish, were evident in part of the root (Supplementary Figure A, black rectangle). Also were sampled parts of root that did not show brownish at all (Supplementary Figure A, red rectangle). Confocal microscopy of FDA-stained roots revealed that, the fragment roots near the point where the pathogen hyphae contacted the root surface showed a bright red fluorescence indicating necrotic tissue (Supplementary Figure B-D). On the other hand, root area without brownish was bright green fluorescence which is consistent with fluorescein localized in the cytoplasm and nucleoplasm of viable plant cells (Supplementary Figure E-G).

These results show that under the same conditions, the root brownish areas, adjacent to the point of entry of the pathogen are dead cells. On the contrary, in the same root, but in areas distant from the entry of the pathogen, cells look normal and are alive.

Z. Xia 1995. Staining Methods for Cell Death. Gladstone Institute

<http://gladstone.org/u/sfinkbeiner/labdocs/cytochemtech/Stain.method.cell.death.pdf>

Supplementary Figure

Effects of *Fusarium virguliforme* on the viability of root cells. **A:** Roots from soybean plantlets cultivated *in vitro* in modified Strullu-Romand (MSR) medium observed by naked eyes. The plants were inoculated with a plug of PDA (25 mm^2^) covered by *Fusarium virguliforme (Fv).* The black rectangle shows root symptoms, 72 h post-inoculation. The red rectangle shows uninfected roots (no symptoms). Bar = 1cm. The roots were stained with FDA-PI. Green and red fluorescence exhibit viable and dead root cells, respectively. **B-D:** Confocal image showing dual staining with FDA-PI in fragment of soybean root with “necrotic” symptoms (in black rectangle). **E-G:** Confocal image showing dual staining with FDA-PI in fragment of uninfected soybean root (no symptoms, red box) Bar = 50 µm.
