# Supplementary figures and images for: An In Vitro Method for Studying the Three-Way Interaction between Soybean, Rhizophagus irregularis and the Soil-Borne Pathogen Fusarium virguliforme
Source: Front Plant Sci. 2017 Jun 16;8:1033. doi: 10.3389/fpls.2017.01033 (PMC5472683; doi:10.3389/fpls.2017.01033)

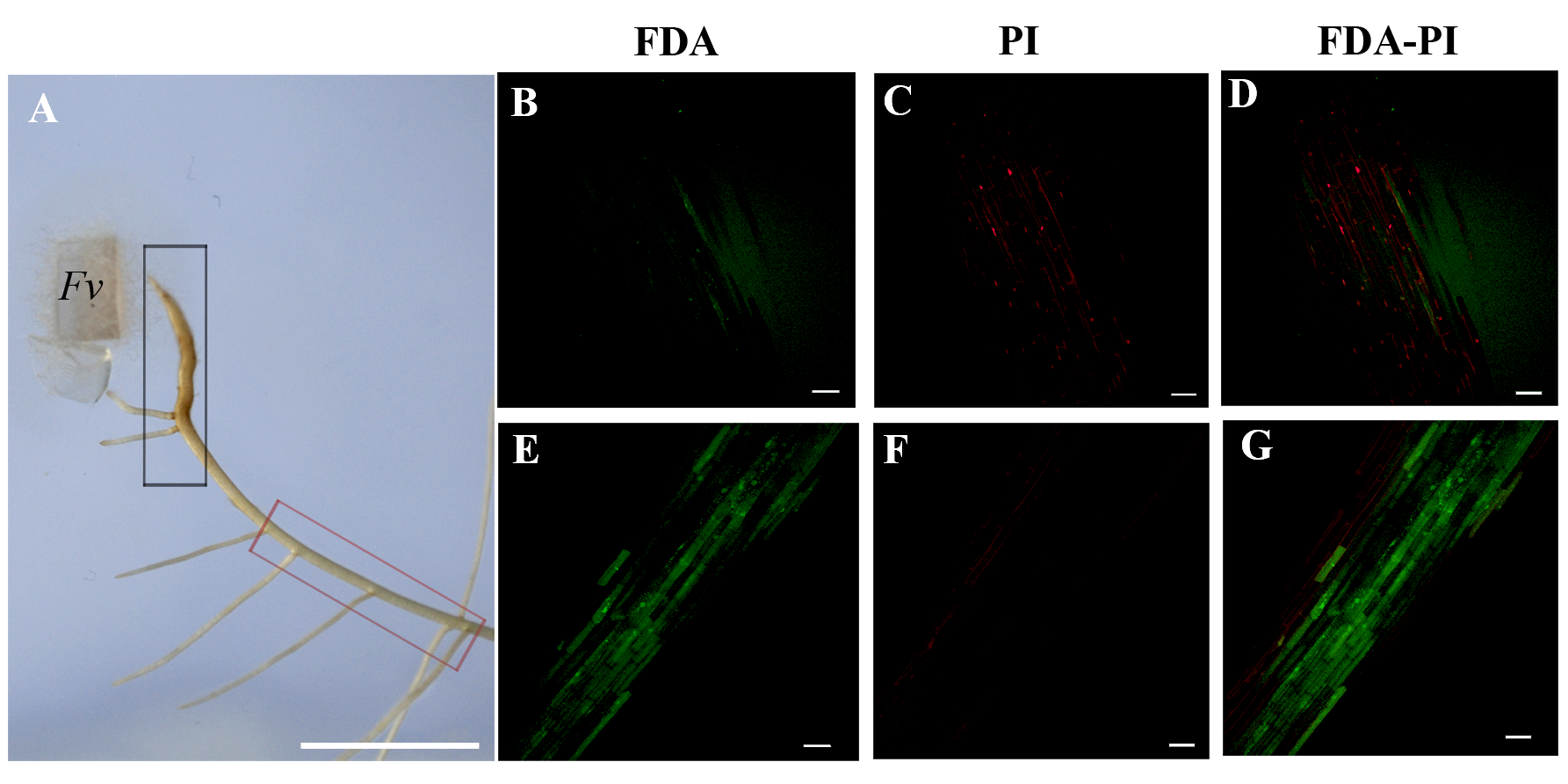

Supplement: Supplementary file 2 [file Image_1.TIF]
